# Primary progressive aphasia: in search of brief cognitive assessments

**DOI:** 10.1093/braincomms/fcac227

**Published:** 2022-09-06

**Authors:** Jordi A Matias-Guiu, Stephanie M Grasso

**Affiliations:** Department of Neurology, Hospital Clínico San Carlos, Health Research Institute ‘San Carlos’ (IdISCC), Universidad Complutense de Madrid, Madrid, Spain; Department of Speech, Language and Hearing Sciences, University of Texas, Austin, TX, USA

## Abstract

This scientific commentary refers to ‘Utility of the Addenbrooke’s Cognitive Examination III online calculator to differentiate the primary progressive aphasia variants’ by Foxe *et al*. (https://doi.org/10.1093/braincomms/fcac161) and ‘A “Mini Linguistic State Examination” to classify primary progressive aphasia’ by Patel *et al*. (https://doi.org/10.1093/braincomms/fcab299)

**This scientific commentary refers to ‘Utility of the Addenbrooke’s Cognitive Examination III online calculator to differentiate the primary progressive aphasia variants’ by Foxe *et al*. (https://doi.org/10.1093/braincomms/fcac161) and ‘A “Mini Linguistic State Examination” to classify primary progressive aphasia’ by Patel *et al*. (https://doi.org/10.1093/braincomms/fcab299)**

Primary progressive aphasia (PPA) is a clinical syndrome characterized by a progressive and predominant impairment of language function due to an underlying neurodegenerative process. Over the last few decades, knowledge surrounding the nature and treatment of this syndrome has notably expanded. Accurate diagnosis by variant is important in both research and clinical settings because classification into the three main clinical variants of PPA (non-fluent/agrammatic, semantic and logopenic aphasia) has improved knowledge regarding clinicopathological associations. Current diagnostic consensus criteria suggest the use of several tasks to assess patients with PPA in order to achieve a correct classification by variant.^1^ However, diagnosis by variant is frequently challenging and relies upon the outcomes of comprehensive cognitive and language assessment conducted by multidisciplinary teams, often coupled with neuroimaging and biomarker findings. Cognitive-linguistic assessments are often time-consuming, based on batteries initially developed for non-neurodegenerative causes such as stroke-induced aphasia, or consist of in-house protocols.^2^ In addition, standardized cognitive assessments commonly used in amnestic forms of dementia are not adequate for patients with PPA due to the predominant language symptoms that are characteristic of this syndrome. Thus, there is a clinical need for brief instruments designed to evaluate patients with PPA. These instruments should be constructed such that clinicians and researchers are able to utilize efficient means to determine a diagnosis of PPA and its variants. Additionally, such measures should show adequate psychometric and diagnostic properties and should also serve to monitor progression of symptoms.

In this area of investigation, two major advances have been recently published in *Brain Communications*. First, Patel *et al*.^3^ describe the Mini Linguistic State Examination (MLSE) and present the first validation study of the MLSE in a cohort of 54 patients with PPA. The MLSE is a brief test that consists of 11 subtests focused on speech and language. Five error types are registered, which are used to generate a profile of impairment according to the following domains: motor speech, semantic knowledge, phonology, syntax and verbal working memory. The authors obtained high levels of internal consistency and good areas under the curve. In addition, a random forest algorithm was trained and validated, obtaining diagnostic accuracies of 92–98%. A Spanish version has already been developed and validated in PPA,^4^ and many other language and cultural adaptations are currently underway.

In addition, Foxe *et al*.^5^ recently investigated the utility of the third version of Addenbrooke’s Cognitive Examination (ACE-III) to differentiate the PPA variants. The authors created an automatic calculator based on individual item analysis. The development of the interactive calculator was initially based on an analysis of 90 patients with PPA and 104 healthy controls. The initial results were further validated in an independent sample of 49 patients with PPA, which also demonstrated high sensitivity. The ACE-III is a brief test, initially developed for the assessment of frontotemporal dementia and related disorders, that examines five main cognitive domains: attention and orientation, memory, verbal fluency, language and visuospatial abilities. The test has now been adapted and validated in >30 languages.

In addition to the two aforementioned instruments, other interesting tools for the rapid screening of PPA have emerged in recent years. These screening tools include the Progressive Aphasia RatIng Scale (PARIS)^6^ a tool specifically designed for PPA, and the Dépistage Cognitif de Québec,^7^ which is a more general cognitive test for assessment of atypical dementia. Additionally, measures for capturing the presence/severity of language symptoms in neurodegenerative disease have also been recently developed including the Progressive Aphasia Language Scale (PALS), Progressive Aphasia Screening Scale (PASS) and the Screening for Aphasia in NeuroDegeneration Battery (SAND).

Taken together, there have been recent efforts to develop clinically useful screening and assessment tools in the realm of PPA. Future studies should compare the diagnostic capacity between instruments to evaluate the optimal assessment tool for each clinical purpose or setting (e.g. early diagnosis, monitoring, etc.). However, it is worth mentioning that these tools could be combined in order to provide complementary information regarding a given patient’s profile. The ACE-III includes several language and non-language items, which is useful for establishing a cognitive profile.^8^ Conversely, the MLSE is focused on language, which could provide a more detailed assessment of language characteristics, but gathers no information about other cognitive functions, which are often involved in PPA, especially with disease progression. Furthermore, the ACE-III is scored using the accuracy of the responses, while the MLSE focuses on error types. Thus, each brief test provides unique information when assessing PPA patients and may guide clinical decision-making regarding treatment planning.

Another interesting aspect of these studies is that the field is moving towards the development and application of automatic tools for PPA diagnosis.^9^ For example, Foxe *et al*.^5^ provided an automated calculator, and Patel *et al*.^3^ trained machine learning algorithms and proposed a decision tree for guiding PPA diagnosis. Machine learning techniques may be a promising method in the identification of subgroups, by predicting diagnoses using test scores and selecting the most sensitive test items, thereby reducing the length of evaluations and ultimately moving towards computer-aided diagnosis.^10^ In addition, new technologies could extract qualitative features or provide a more in-depth analysis of spontaneous speech, which could also be useful in improving the diagnosis of language disorders.

The studies mentioned above report novel tools for the assessment of patients with PPA. Importantly, these tools were developed specifically for the assessment of patients with PPA and/or neurodegenerative disorders. These novel tools provide the foundation for future adaptations of these tests to other languages and sociocultural contexts which may serve to promote multicentre studies enrolling larger and more culturally and linguistically diverse samples. The development and broader inclusion of common instruments specifically designed for PPA represents a crucial first step towards improving diagnosis and disease monitoring for patients with PPA. Lastly, such measures may be used in future clinical trials to capture the potential improvement of PPA-specific symptoms following targeted interventions.

## Data Availability

Data sharing is not applicable to this article as no new data were created or analysed.
